# Stener-Like Lesion of the Radial Collateral Ligament of the Thumb Metacarpophalangeal Joint

**DOI:** 10.31486/toj.25.0076

**Published:** 2026

**Authors:** Alexander Crowley, Jakob Oury, Katherine Fisher, Carol Meyer

**Affiliations:** ^1^ Department of Orthopedics, Ochsner Clinic Foundation, New Orleans, LA; ^2^ The University of Queensland Medical School, Ochsner Clinical School, New Orleans, LA

**Keywords:** *Collateral ligaments*, *finger injuries*, *hand injuries*, *joint instability*, *metacarpophalangeal joint*, *thumb*

## Abstract

**Background:**

Stener lesions involve rupture of the ulnar collateral ligament of the thumb metacarpophalangeal joint with displacement of the ligament superficial to the adductor aponeurosis, preventing healing. Rarely, however, entrapment of the ligament by the overlying abductor pollicis brevis aponeurosis can lead to a Stener-like lesion. Few Stener-like lesions have been described, and surgical repair is recommended.

**Case Report:**

A 27-year-old female fell while skiing and hyperextended her right thumb. She presented with thumb pain, swelling, ecchymosis, and laxity. X-ray showed subluxation of the metacarpophalangeal joint. Magnetic resonance imaging demonstrated a complete radial collateral ligament tear with the proximal end displaced superficial to the abductor pollicis brevis aponeurosis (Stener-like), blocking reduction. An incision was made over the metacarpophalangeal joint. The radial collateral ligament fibers were interposed in the abductor pollicis brevis aponeurosis. Metacarpophalangeal joint arthrotomy revealed volar plate interposition that was removed to restore metacarpophalangeal joint reduction. The radial collateral ligament was repaired with a modified Kessler technique and augmented using an internal suture brace involving a suture anchor reinforced with suture tape for collateral metacarpophalangeal joint support. The abductor pollicis brevis aponeurosis was repaired, the joint was confirmed stable, and the wound was closed and dressed in a thumb spica splint.

**Conclusion:**

This case describes a rare Stener-like lesion of the thumb radial collateral ligament, adding to the limited literature on this injury pattern. Such lesions are far less common than the classic Stener lesion. Early recognition is critical because interposed tissue will block reduction and healing.

## INTRODUCTION

Thumb collateral ligament tears are common, but ulnar collateral ligament tears occur far more frequently than radial collateral ligament tears. One study of 1,000 thumb injuries found that ulnar collateral ligament tears may be involved in up to 86% of thumb injuries,^1^ while radial collateral ligament tears are estimated to constitute only 10% to 42% of thumb collateral ligament injuries.^2^ A classic Stener lesion involves distal ulnar collateral ligament stump displacement superficial to the adductor aponeurosis.^3^ In contrast, radial collateral ligament Stener-like lesions, in which the torn radial collateral ligament stump is interposed superficial to the abductor aponeurosis, are rare, as the abductor pollicis brevis tendon inserts broadly and covers the distal insertion of the radial collateral ligament.^4^

As with Stener lesions, early recognition of soft tissue interposition in a Stener-like lesion is critical, as nonoperational treatment may fail.^5^ We present a case of a Stener-like lesion of the right thumb radial collateral ligament that required surgical repair.

## CASE REPORT

While skiing on March 13, 2025, a 27-year-old, right-hand-dominant female fell and hyperextended her right thumb. She noted immediate pain and swelling. On March 17, 2025, she presented to clinic with notable swelling, ecchymosis, and decreased range of motion of the right metacarpophalangeal joint. She denied any numbness, tingling, or prior thumb injuries. Her medical history—asthma and wisdom tooth extraction—was not relevant to the injury.

Stress testing revealed laxity of the radial collateral ligament compared to the uninjured left thumb. No gross deformity was noted.

**Figure 1.**
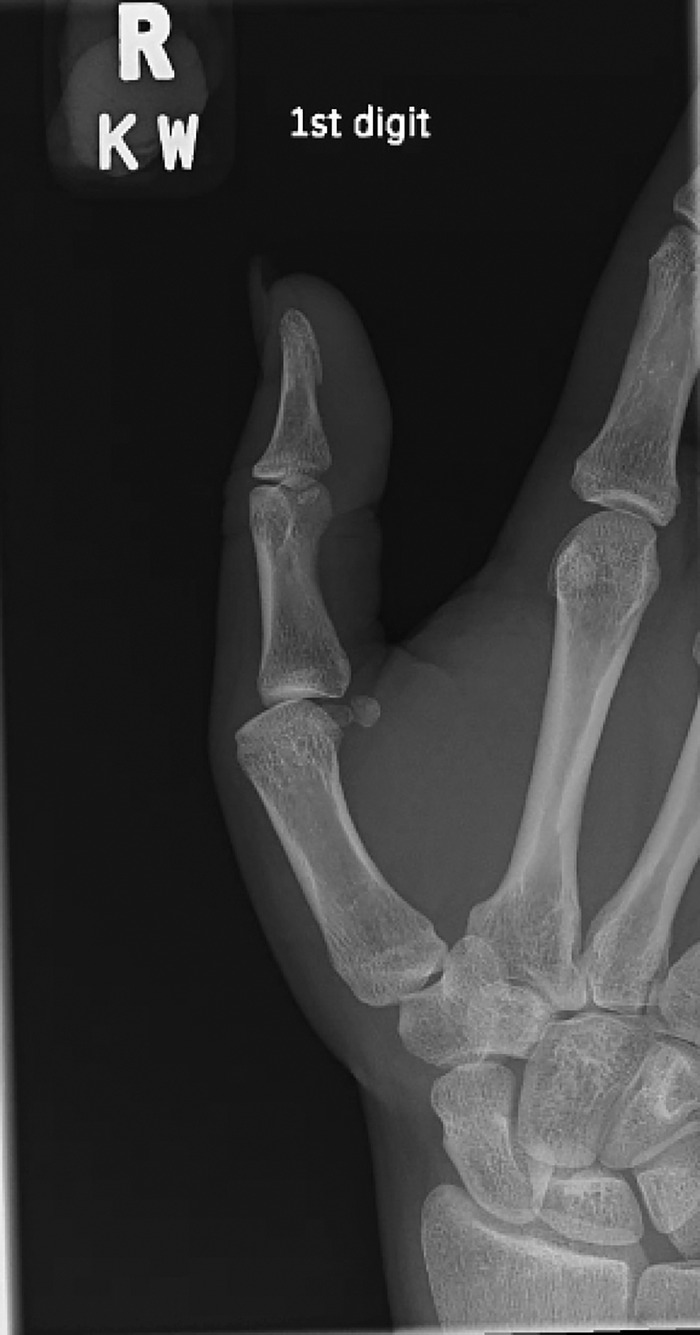
Preoperative radiograph of the right thumb shows subluxation of the metacarpophalangeal joint.

**Figure 2.**
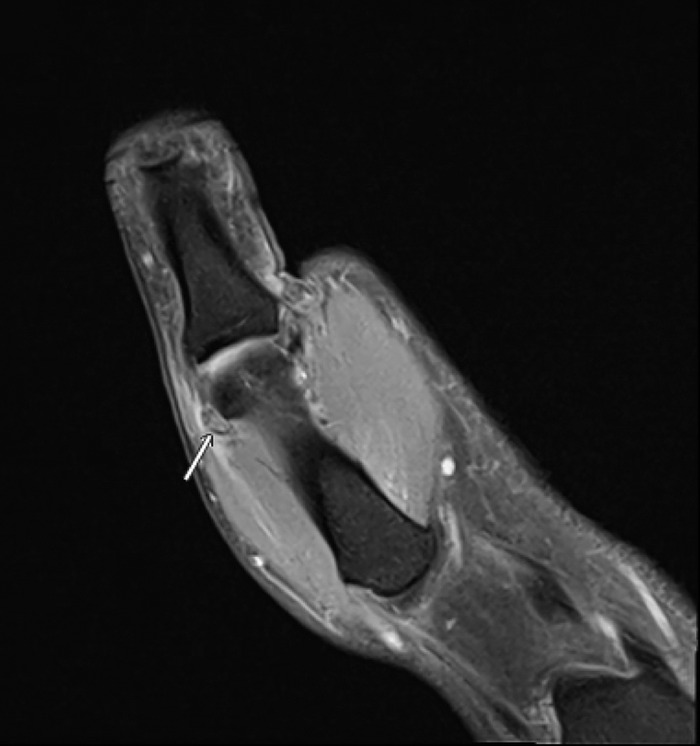
Preoperative magnetic resonance imaging of the right thumb shows the proximal radial collateral ligament tear with soft tissue interposition into the abductor pollicis brevis aponeurosis (arrow).

Plain radiographs showed subluxation of the metacarpophalangeal joint but no fracture (Figure 1). Magnetic resonance imaging (MRI) demonstrated a complete tear of the proximal radial collateral ligament at the metacarpal attachment, with the torn fibers displaced superficial to the abductor aponeurosis, consistent with a Stener-like lesion (Figure 2). Additional findings included a partial volar plate tear proximally with interposition, mild metacarpophalangeal joint subluxation, and possible partial ulnar collateral ligament sprain.

**Figure 3.**
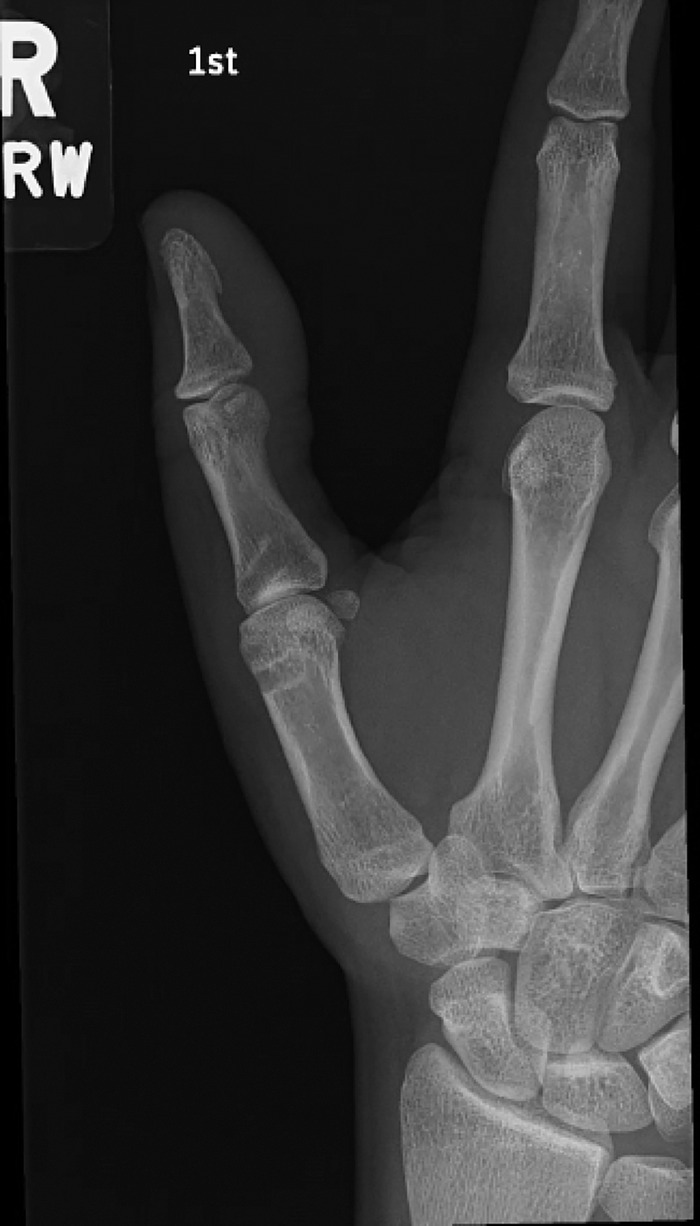
Postoperative radiograph of the right thumb shows restored alignment of the metacarpophalangeal joint.

Surgery was performed on March 20, 2025. A radial incision was made over the metacarpophalangeal joint. The abductor aponeurosis was incised to expose the radial collateral ligament, which was found to be frayed and retracted superficial to the abductor aponeurosis, confirming a Stener-like lesion. Metacarpophalangeal joint arthrotomy revealed interposed volar plate that prevented reduction of the joint. A Freer instrument was used to remove tissue from the joint to allow reduction. Nonabsorbable braided polyethylene suture was used to repair the intrasubstance radial collateral ligament tear in a modified Kessler manner. Persistent joint laxity necessitated augmentation with an internal suture brace construct. A guidewire was driven into the radial collateral ligament origin on the metacarpal head, and a second guidewire was driven into the proximal phalanx base slightly volar to midline. X-ray was used to check anchor placement and joint reduction. A knotless suture anchor loaded with suture tape was placed into the proximal phalanx base; the limbs of the suture tape were then used to re-create the proper and accessory collateral ligaments. The proximal phalanx was held reduced on the metacarpal head and the second anchor was used to secure the reconstruction into the metacarpal head. Intraoperative fluoroscopy revealed no subluxation of the proximal phalanx and no gapping of the radial collateral ligament reconstruction or joint. Postoperative radiographs showed the reduced metacarpophalangeal joint (Figure 3).

Postoperatively, the patient wore a thumb spica splint and progressed through supervised therapy 3 times per week. At 12-week follow-up, she reported no pain, minimal residual weakness, and full return to daily activities. She weaned off the thumb spica splint during the subsequent 2 weeks.

## DISCUSSION

The metacarpophalangeal joint of the thumb relies on collateral ligaments—the ulnar collateral ligament and radial collateral ligament—to provide stability during pinch and grasp activities. The radial collateral ligament lies deep to the abductor pollicis brevis tendon, originates from the dorsal aspect of the first metacarpal head, and inserts onto the proximal phalanx base, working in conjunction with the accessory collateral ligament and surrounding extensor and abductor aponeurosis to resist varus stress.^6^ An anatomic study of the thumb metacarpophalangeal joint from 2008 posits that the broad, fibrous abductor pollicis brevis tendon makes displacement of the ligament into a superficial tissue plane impossible.^6^ Another mechanism thought to combat tissue superposition, in contrast to Stener lesions, is the different incidence of distal ligament ruptures. A 2019 anatomic study suggests that only distal ligament rupture can produce a sufficiently long proximal stump to permit mechanical displacement; however, distal tears represented only 29% of radial collateral ligament injuries in a single-institution cohort of 38 patients, compared with 90% of ulnar collateral ligament ruptures.^4,7^ This point further highlights the rarity of the case we present, as the Stener-like lesion in our patient involved the proximal end of the ligament.

Radial collateral ligament tears with suspected entrapment and laxity without a definite endpoint warrant early MRI to assess for tissue superposition.^5^ Failure to recognize this pattern may result in persistent instability and poor outcomes with nonoperative care.^8^ In our case, surgical exploration confirmed the Stener-like entrapment and the necessity of removing volar plate interposition to restore joint reduction. An internal suture brace construct provided the additional stability necessary because of the frayed radial collateral ligament fibers and persistent laxity. This technique may be valuable when repair alone is insufficient. Other suture-based techniques and autologous graft reconstruction have been described in the literature as useful ways to augment radial collateral ligament repair.^9^

## CONCLUSION

Radial collateral ligament Stener-like lesions, although rare, have a high risk for conservative management failure. Prompt surgical repair and adjunct stabilization may be required, with the awareness that soft tissue interposition can occur on both the ulnar and radial sides of the thumb metacarpophalangeal joint.

## Notes

*This article meets the Accreditation Council for Graduate Medical Education and the American Board of Medical Specialties Maintenance of Certification competencies for Patient Care and Medical Knowledge.*
